# Has the introduction of the ‘2 week rule’ in the UK led to an earlier diagnosis of urological malignancy?

**DOI:** 10.3332/ecancer.2011.215

**Published:** 2011-08-31

**Authors:** N Vasdev, AC Thorpe

**Affiliations:** Department of Urology, Freeman Hospital, Newcastle upon Tyne, UK

## Abstract

**Introduction:**

The ‘2 week wait’ target for haematuria assessment has been recommended for early diagnosis of urological cancer. We compare our cancer detection rates pre- and post-introduction of this target and its impact on stage at diagnosis.

**Patients and methods:**

A total of 1,740 patients were enrolled prospectively in the study at our units ‘one stop fast track haematuria clinic’ between April 2003 and March 2006. Evaluation consisted of basic demographics, history and examination, routine blood tests, urinalysis, urine culture and urine cytology. All patients underwent a renal ultrasound, IVU (intravenous urogram) and flexible cystoscopy. The detection rate of urological malignancy was compared to a previous cohort at our institution (April 1994 to March 1997).

**Results:**

A total of 1,067 males and 673 females with a mean age of 60.8 years (range 16–96) were included in the study. The overall cancer detection rate was 18%. With the introduction of the ‘2 week rule’ referrals, we noted a 57% increase in the detection of urological malignancies while comparing previous published data from our institution. There was no statistical significance in stage at presentation following the introduction of the ‘2 week rule’.

**Conclusion:**

Patients with haematuria need to be investigated promptly. The ‘2 week rule’ has increased the cancer detection rate in our institution.

## Introduction

Gross haematuria is common with a community prevalence of 2.5% [1] and estimated to account for 4% to 20% of all urological hospital visits [2]. The gradual increase in patient referrals to urological clinics is attributed to the increased use of urine dipstick analysis in the community [3]. Current recommendations in the United Kingdom recommend investigations of haematuria urgently via a ‘Fast track one stop haematuria clinic’ [4]. The economic burden of investigating haematuria is increased if patients with no significant pathological condition continue to be followed [5]. In 2000, we had published a paper from our department [6] analysing haematuria data on 1,930 patients evaluating current diagnostic practices.

We compare data from a previous publication from our department [5] evaluating changes in urological malignancy staging with the introduction of UK government ‘2 week rule’ patients. Additionally we evaluate the impact of the ‘2 week rule’ on the stage at presentation of urological cancers.

## Patients and methods

We prospectively analyse data from 1,740 patients who attended a ‘one stop fast track haematuria clinic’ at the Freeman Hospital between April 2003 and March 2006. Evaluation consisted of basic demographics, history and examination, routine blood tests, urinalysis and cytology. In addition patients underwent a renal ultrasound, IVU (intravenous urogram) and flexible cystoscopy. We compare data from a previous published paper [5] in our unit assessing the effect of the ‘2 week rule’ in stage migration of urological cancer at initial diagnosis.

## Results

Mean age of presentation with haematuria was 61.7 years (range 17–96) in 1,067 males and 59.9 years in 673 females (range 16–94). The degree of haematuria ranged from asymptomatic dipstick haematuria (24.1%), symptomatic dipstick haematuria (20.7%), gross painless haematuria (38.3%), gross painful haematuria (16.3%), and haematospermia/microscopic haematuria (0.5%) (Table 1). A total of 44 patients (2.5%) had been previously investigated for haematuria with no previous cause identified.

Final diagnosis in 1,740 patients with varying degrees of haematuria is shown in Table 2. In 27% (*n* = 470) no cause of haematuria was found. Renal tumours including upper tract transitional cell carcinoma (TCC) was found in 39 patients (13 females and 26 males), bladder cancer in 243 patients (61 females and 182 males), and urinary tract stones in 90 patients (40 females and 50 males). Four-hundred and thirty eight patients were diagnosed to have a urinary tract infection either on initial referral or during investigation in the haematuria clinic. Fifty-two patients (30 female and 22 male) were diagnosed with nephrological disease.

Of the 1,740 referrals received 62.4% (*n* = 1,087) were ‘2 week rule’ referrals and 37.5% (*n* = 653) were non-urgent. In patients with bladder cancer 87.2%, renal cancer (including upper tract TCC) 89.7%, prostate cancer 48.7%, penile cancer 100% and testicular cancer 100% of referrals were ‘2 week rule’ referrals. 46.1% of patients with no diagnosis on workup in the clinic were ‘2 week rule’ referrals. 46.1% of patients with nephrological disease, 73.6% with a vascular re-growth of prostate following previous TURP (Trans-urethral resection of prostate) with a vascular BPH (Benign prostatic hyperplasia), 55.3% with a vascular BPH (benign prostatic hyperplasia) were referred as ‘2 week rule’ referrals. The type of referral and aetiology of haematuria are analysed in Table 3.

### Has the introduction of the ‘2 week rule’ referrals lead to an increase incidence of urological malignancy diagnosis

With the introduction of the ‘2 week rule’ patients with haematuria should be referred to the ‘haematuria clinic’ for an urgent assessment within 2 weeks. We aim to analyse the incidence of new diagnosis of urological malignancy in patients referred from April 1994 to March 1997 (group 1) to our current group of patients seen from April 2003 to March 2006 (group 2). The total number of patients seen in both groups was similar with 1,930 (group 1) and 1,740 (group 2).

A total of 184 urological malignancies were diagnosed in group 1 and 326 urological malignancies in group 2. This suggests an increase in diagnosis in urological malignancy in group 2 by 57%. 86 more new cases of TCC bladder were diagnosed in group 2. These included 45 new cases with pTa TCC bladder, 16 with T1 TCC bladder, 23 with T2 TCC bladder and 2 T3/T4 TCC bladder. Sixteen more renal cell carcinomas were diagnosed in group 2. 28 more cases of prostate cancer were also diagnosed in group 2. These data suggested an increase in the incidence of more urological cancer diagnosis. While it would appear that there was a higher number of cases diagnosed with superficial bladder TCC in group 2, χ^2^-testing showed no statistical significance between patients presenting with superficial compared to muscle invasive tumours between the two groups (Table 4).

## Discussion

The most important reason for investigating haematuria is to exclude urological malignancies [7,8,9]. The aim of our paper is to evaluate the incidence of urological malignancy in patients presenting to and to assess stage migration following the introduction of the ‘2 week rule’.

The UK Governments white paper *The New NHS* recommends a specialist review for any patient with a suspected malignancy following their initial assessment in primary care [10]. These rules were implemented for patients with haematuria in 2000 [11]. The Department of health has recommended that for a every cancer referral must be marked ‘urgent’ with the mention of ‘cancer’ or include specific requests under the ‘2 week rule’ [17]. The main aim of the ‘2 week rule’ referrals is to offer rapid access for urological patients include patients with painless haematuria, microscopic haematuria, testicular mass, palpable renal mass, raised PSA (prostate-specific antigen) and suspected penile cancer [12,13]. To have an impact on the introduction of the ‘2 week rule’ patients must be diagnosed earlier with a stage migration in cancer. We compare the details of two groups of referrals during the era where there were no ‘2 week rule’ (*N* = 1,930, April 1994 to March 1997) versus the era of introduction of the ‘2 week rule’ referrals (*N* = 1,740, April 2003 to March 2006). Our data suggest a 57% increase in the incidence of urological malignancy with the introduction of the ‘2 week rule’. The marked increase in the diagnosis of non muscle invasive TCC of bladder by 45 cases in the pTa group and 23 cases in the muscle invasive group. Sixteen more cases of renal cell carcinomas were diagnosed and 28 more cases of prostate cancer. These data confirm the importance of investigating all patients with haematuria urgently.

The incidence of urological cancers picked up following the introduction of the ‘2 week rule target’ in our cohort was higher than the observed trend noted in the UK. Although there was no statistical difference of stage migration of bladder cancers, it is recognised that earlier presentation leads to better outcome. For instance, Hollenbeck *et al.* recently showed that a delay in the diagnosis of bladder cancer increased the risk of death from disease independent of tumour grade and or disease stage. They looked at nearly 30,000 patients with bladder cancer diagnosis and found that patients who had a diagnosis delay of 9 months were more likely to die from bladder cancer compared with patients who were diagnosed within 3 months with the risk not markedly attenuated after adjusting for disease stage and tumour grade In that study, the effect was strongest among patients who had low-grade tumours and low-stage disease [14].

There has been some debate about the usefulness of the ‘2 week wait’ target in urology and other specialties.

A prospective study of the ‘2 week wait’ haematuria clinic found that some current guidelines are leading to referral of patients with low cancer risk and recommended that review of the system was vital to allow the appropriate detection of malignancy without overburdening the premium clinic slots with the healthy [15]. A large prospective cohort of patients referred to the breast clinic found that number of cancers detected in the 2-week wait population is decreasing, and an unacceptable proportion is now being referred via the routine route [16]. In colorectal surgery, it was found that the inherently poor specificity of the system has resulted in a poor (and decreasing) cancer detection rate and a steadily growing volume of the hospital referrals. The system has been shown to have an adverse impact on the waiting times for routine colorectal referrals [17].

In conclusion, all patients with haematuria should be examined promptly. The ‘2 week rule’ has lead to an increase incidence of urological malignancy diagnosis although no statistically significant stage migration was noted.

## Figures and Tables

**Table 1:** Details of initial referral with haematuria in 1,740 patients

**Type of haematuria**	**N**	**%**	**‘2 week rule’ referral**	**Non-urgent referral**
Asymptomatic dipstick	420	24.1 %	227	193
Symptomatic dipstick	361	20.7 %	181	180
Gross painless haematuria	664	38.3 %	493	171
Gross painful haematuria	285	16.3 %	181	104
Haematospermia/microscopic haematuria	10	0.5 %	5	5

**Table 2:** Diagnosis of 1,740 patients evaluated for haematuria

**Diagnosis**	**All patients (N)**	**All patients (%)**	**No. of females (N)**	**No. of females (%)**	**No. of males (N)**	**No. of males (%)**
No diagnosis	470	27.01%	253	37.59%	217	20.33%
Renal carcinoma (including TCC)	39	2.24%	13	1.93%	26	2.40%
Bladder cancer	243	13.96%	61	9.06%	182	17.05%
Prostate cancer	39	2.23%	0	0%	39	3.65%
Penile cancer	1	0.05%	0	0%	1	0.09%
Gynaecological cancer	4	0.22%	4	0.59%	0	0%
Testicular cancer	1	0.05%	0	0%	1	0.09%
Nephrological cause	52	2.98%	30	4.40%	22	2.06%
UTI	438	25.17%	227	33.70%	206	25.75%
Previous TURP	72	4.13%	0	0%	72	6.74%
Vascular BPH	94	5.40%	0	0%	94	8.80%
Urinary tract calculi	90	5.17%	40	5.94%	50	4.68%
Benign causes	197	11.32%	45	6.68%	157	14.71%
Total	1,740	100%	673	100%	1,067	100%

**Table 3:** Type of initial referral and aetiology of haematuria as diagnosed in the haematuria clinic

**Diagnosis**	**All patients (N)**	**‘2 week rule’ referrals (N)**	**‘2 week rule’ referrals (%)**	**Non-urgent referrals (N)**	**Non-urgent referrals (%)**
No diagnosis	470	217	19.96%	253	38.74%
Renal carcinoma (including TCC)	39	35	3.21%	4	0.61%
Bladder cancer	243	212	19.50%	31	4.74%
Prostate cancer	39	19	1.74%	20	3.06%
Penile cancer	1	1	0.09%	0	0.00%
Gynaecological cancer	4	4	0.36%	0	0.00%
Testicular cancer	1	1	0.09%	0	0.00%
Nephrological cause	52	24	2.20%	28	4.28%
UTI	438	280	25.75%	178	27.25%
Previous TURP	72	53	4.87%	19	2.90%
Vascular BPH	94	52	4.78%	42	6.43%
Urinary tract calculi	90	53	4.87%	37	5.66%
Benign causes	197	136	12.51%	41	6.27%
Total	1,740	1,087	100%	653	100%

**Table 4:** Comparison of incidence of urological malignancy between patient referrals in group 1 (patient referred from April 1994 to March 1997) and group 2 (April 2003 to March 2006)

**Type of Urological Malignancy**	**Group 1 (April 1994 to March 1997)**	**Group 2 (April 2003 to March 2006)**
T_a_ TCC bladder	80	129
T_1_ TCC bladder	47	63
T_2_ TCC bladder	6	29
T_3_/T_4_ TCC bladder	20	22
Renal cell carcinoma	12	28
TCC renal pelvis	4	4
Ureteric TCC	4	7
Adenocarcinoma of prostate	11	39
